# Replacement of aneurysmal right coronary artery with reconstruction of posterior descending and right ventricular branches in coronary artery fistula draining into the coronary sinus: a case report

**DOI:** 10.1093/ehjcr/ytaf269

**Published:** 2025-05-28

**Authors:** Yuya Yamazaki, Hiroyuki Nakajima, Itaru Igarashi, Takeshi Arai, Daichi Takagi

**Affiliations:** Department of Cardiovascular Surgery, Akita University Graduate School of Medicine, 1-1-1, Hondo Akita City, Akita 010-8543, Japan; Department of Cardiovascular Surgery, Akita University Graduate School of Medicine, 1-1-1, Hondo Akita City, Akita 010-8543, Japan; Department of Cardiovascular Surgery, Akita University Graduate School of Medicine, 1-1-1, Hondo Akita City, Akita 010-8543, Japan; Department of Cardiovascular Surgery, Akita University Graduate School of Medicine, 1-1-1, Hondo Akita City, Akita 010-8543, Japan; Department of Cardiovascular Surgery, Akita University Graduate School of Medicine, 1-1-1, Hondo Akita City, Akita 010-8543, Japan

**Keywords:** Case report, Coronary artery fistula, Coronary aneurysm, Right coronary artery, Right ventricular branch

## Abstract

**Background:**

Dilated coronary arteries associated with coronary fistulas can cause late complications, such as rupture and myocardial ischaemia due to intraluminal thrombosis and distal embolism. This case report presents the successful surgical treatment of a coronary artery fistula causing a tortuous and dilated right coronary artery (RCA).

**Case summary:**

A 69-year-old female presented with congestive heart failure and atrial fibrillation detected on the electrocardiogram. Chest roentgenography showed lung congestion and cardiomegaly, with a cardiothoracic ratio of 84%. Echocardiography revealed severe mitral and tricuspid regurgitation and pulmonary hypertension. Coronary angiography revealed a dilated RCA fistula draining into the coronary sinus, with a Qp/Qs ratio of 2.3. Surgery was performed via median sternotomy. Under cardiac arrest, mitral valve replacement and tricuspid valve plasty were performed. The RCA was divided at the proximal portion, just distal to the origin of the conus branch. Through a longitudinal incision on RCA, the orifices of the posterior descending artery and the two major right ventricular branches were identified and trimmed as buttons. The dilated RCA was replaced with the saphenous vein graft, which was sequentially anastomosed with the buttons. Postoperative angiography showed visualization of the saphenous vein and right ventricular branches, with the elimination of the huge shunt flow from the RCA.

**Discussion:**

Eliminating shunt flow, resecting the dilated portion of the RCA > 10 mm in diameter, and recreating sufficient coronary perfusion to the right ventricle by replacement of RCA are crucial for favourable early and late clinical outcomes of diffusely aneurysmal coronary artery fistula.

## Graphical Abstract

**Graphical Abstract:**
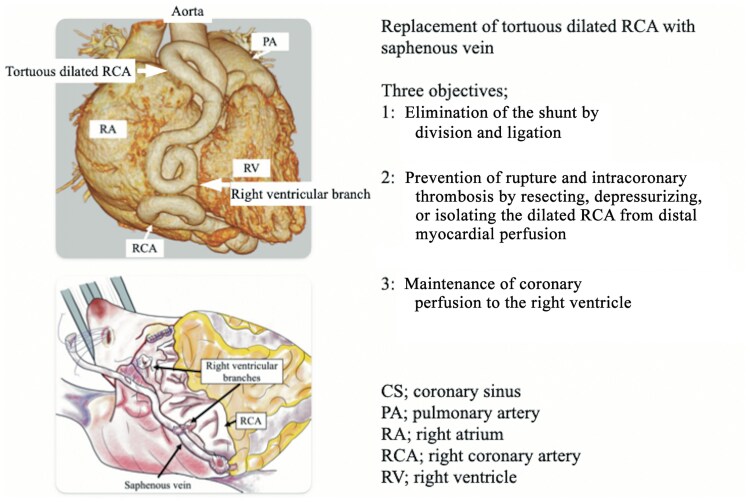

**Learning points**

- Understand the clinical course of coronary artery fistulas leading to heart failure, atrial fibrillation, and valvular disease.
- Recognizing the risk of rupture and intracoronary thrombosis associated with aneurysmal dilatation, even after surgical treatment.
- Identifying the surgical treatment goals for aneurysmal dilatation associated with coronary artery fistulas, including shunt elimination, resection of the dilated portion > 10 mm of the coronary artery, and recreation of myocardial perfusion to the right ventricle.

## Introduction

Coronary artery fistula (CAF), characterized by abnormal communication between the coronary artery and cardiac chambers or other vascular structures, can cause heart failure, valvular disease, pulmonary hypertension, myocardial ischaemia, and aneurysm rupture. Careful planning and timing of the surgical intervention are essential because of the complexity of these conditions.

This case report presents a comprehensive surgical approach for aneurysmal dilatation of the long segment of the right coronary artery (RCA) associated with CAFs.

## Summary figure

**Table:** 

June 2016, 8 years prior to operation	The patient had a systolic murmur at the cardiac apex and was started on medical therapy with a diagnosis of mitral and tricuspid regurgitation concomitant with Basedow’s disease.
February 2017, 7 years prior to operation	Atrial fibrillation and mild mitral regurgitation appeared. The patient had no obvious symptom of heart failure, but valvular disease gradually developed over 6 years.
December 2023, 1 year prior to operation	The patient began experiencing exertional fatigue.
	Echocardiography revealed severe mitral regurgitation with a tricuspid regurgitation pressure gradient of 41 mmHg.
	Coronary computed tomography identified a tortuous and aneurysmal right coronary artery originating from the aorta and converging with the left circumflex artery in the posterior interventricular groove.
November 2024, Day 0	Mitral valve replacement, tricuspid valve repair with papillary muscle approximation, and division and ligation of the fistulas were performed. Two right ventricular branches and posterior descending artery were anastomosed with a saphenous vein graft.
December 2024, Day 20	Coronary Angiography Showed Normalized Coronary Flow To The Right Ventricle And Posterior Descending Artery.

## Case presentation

A 69-year-old woman had been monitored for asymptomatic mitral and tricuspid regurgitation and concomitant Basedow’s disease for 8 years. She recently experienced exertional fatigue and was referred to our hospital for surgical treatment of heart failure. Her blood pressure was 111/53 mmHg, and her heart rate was 67 beats/min. Chest radiography revealed a cardiothoracic ratio of 84%. Echocardiography revealed severe mitral and tricuspid regurgitation, with a tricuspid regurgitation pressure gradient of 41 mmHg.

**Figure 1:**
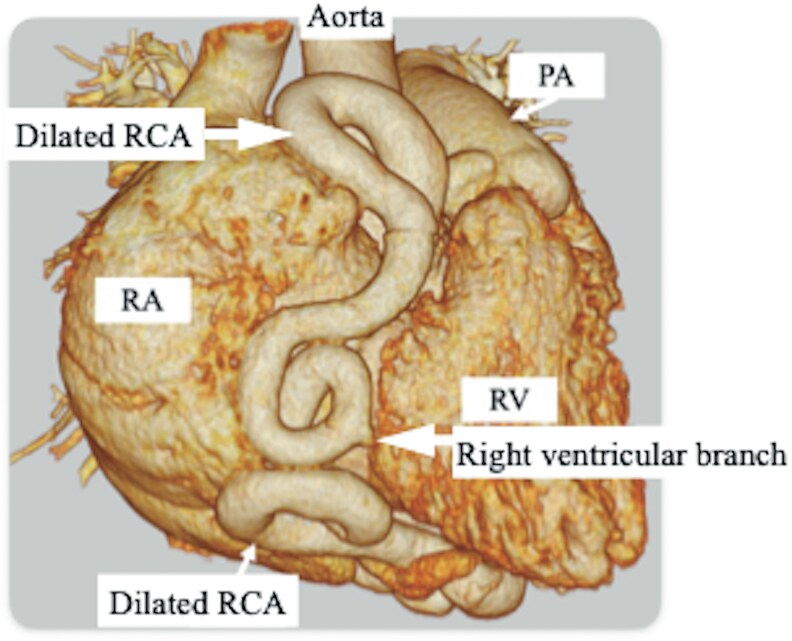
Preoperative computed tomography revealing an aneurysm in the right coronary artery. A small fistula arose from the left circumflex artery as a side branch. Both fistulas drained into the coronary sinus.

Coronary computed tomography angiography revealed two CAFs (*Figure 1*). One was a tortuous aneurysmal RCA coursing anteriorly to the ascending aorta and draining into the coronary sinus. The other was located in the posterior interventricular groove arising from the left circumflex artery. Selective coronary angiography demonstrated aneurysmal dilatation of the RCA with a high flow velocity and a fistula originating from the circumflex artery that drained into the coronary sinus. The RCA was dilated along its entire length, with a diameter ranging from 10 to 16 mm, with a Qp/Qs ratio of 2.3 and a mean pulmonary artery pressure of 28 mmHg. The patient was diagnosed with severe mitral and tricuspid regurgitation, chronic atrial fibrillation, concomitant RCA-to-coronary sinus fistula, and circumflex artery-to-coronary sinus fistula. Our heart team decided to perform surgical treatment.

Surgery was performed via median sternotomy. Cardiopulmonary bypass was performed with ascending aortic cannulation and bicaval drainage. To confirm cardioplegic delivery, a fistula arising from the circumflex artery was ligated at the origin, and the dilated RCA was dissected and ligated distally to the origin of the posterior descending branch during on-pump beating. The ascending aorta was clamped and cardiac arrest was achieved with antegrade and retrograde cardioplegia. The mitral valve exhibited annular dilatation, degeneration, and shortening of the leaflets and was replaced with a 29-mm Epic bioprosthetic valve (Abbott Laboratories, Chicago, IL, USA).

**Figure 2:**
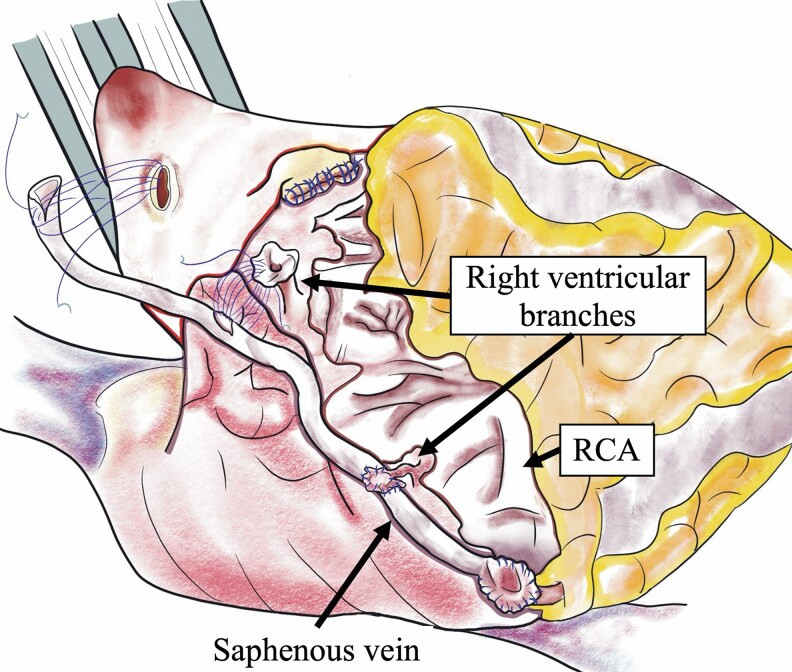
The right coronary artery was divided at the proximal portion and ligated distally to the posterior descending branch. The end of the saphenous vein was anastomosed to the button of the orifice of the posterior descending artery. The buttons of the orifices of two right ventricular branches were sequentially anastomosed to the side of the saphenous vein graft.

The tortuous and dilated RCA was divided at the proximal portion just distal to the origin of the conus branch. Additionally, the RCA was longitudinally incised, and the orifices of the posterior descending artery and the two right ventricular branches were identified and trimmed as buttons. The distal end of the saphenous vein graft was anastomosed to the posterior descending artery. Two buttons for the right ventricular branches were anastomosed to a 4 mm hole on the side of the saphenous vein graft using a 7–0 Prolene® suture (*Figure 2* and Supplementary material online, *Video*). Finally, the left atrial appendage was excised and tricuspid annuloplasty concomitant with papillary muscle approximation was performed.

**Figure 3:**
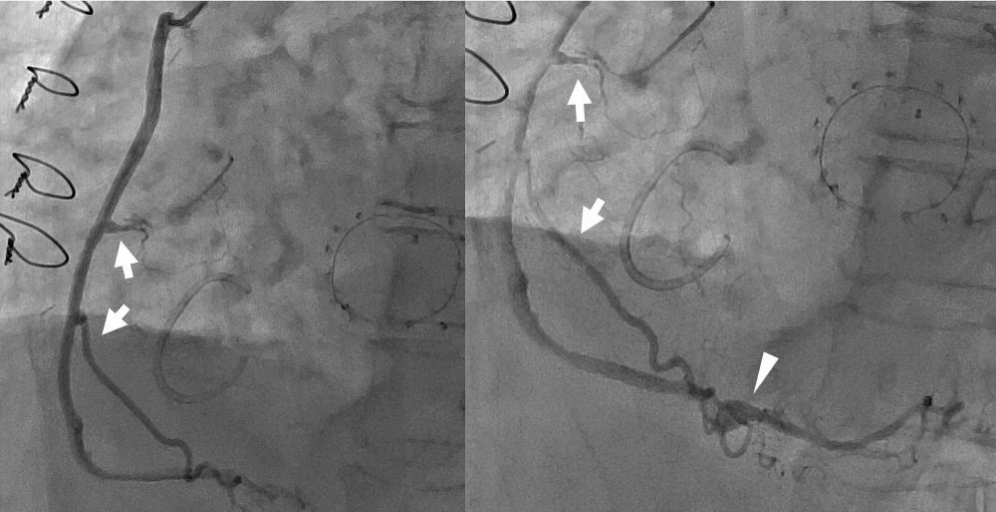
Postoperative angiography showing coronary perfusion maintained to the right ventricle via the conus branch, two right ventricular branches (arrows), and the posterior descending artery (arrowhead) anastomosed with the saphenous vein. Two right ventricular branches were selected based on the size of the orifice and location. Shunt flow from the right coronary artery was completely eliminated, with no aneurysmal dilatation or blind pouch, minimizing the risk of intracoronary thrombosis or rupture.

The patient was extubated the next day and rehabilitation was initiated. Two weeks postoperatively, the patient developed a cardiac tamponade, which was managed with surgical drainage. On postoperative angiography, two right ventricular and posterior descending branches were clearly visualized from the saphenous vein (*Figure 3*). The patient was discharged in a stable condition.

## Discussion

CAFs are occasionally associated with aneurysms, with the incidence of aneurysm formation increasing with age.^1^ When the aneurysm is present in an abnormal fistula vessel arising as a side branch from a coronary artery, ligation or closure at the origin and the distal portion can prevent rupture, eliminate shunting, and improve myocardial perfusion distally. However, when aneurysmal dilatation occurs in the coronary artery, similar to the RCA in the present case, ligation or closure of the fistula alone can cause myocardial ischaemia and thrombosis.^2^ Importantly, a fistula draining into the coronary sinus is significantly associated with late complications, including cardiomyopathy after fistula closure.^3,4^ To achieve appropriate myocardial perfusion, concomitant coronary artery bypass grafting to the distal coronary branch^5^ or interposition using a vein graft^6^ or a covered stent using a catheter approach^7^ can be an effective option.

Surgical management of a dilated coronary artery is crucial to prevent its rupture and intracoronary thrombosis and to guide postoperative antithrombotic therapy. Wauthy *et al*.^8^ reported successful conservative treatment of a dilated coronary segment. In paediatric patients, ligation or occlusion of only the most distal segment is an option for myocardial perfusion.^9^ Conversely, Bauer *et al*.^10^ cautioned regarding spontaneous rupture of an abnormal fistula vessel without aneurysmal dilatation, and Nakahira *et al*.^11^ reported that a residual dilated portion can be ruptured following surgical closure of the fistula. Valente *et al*.^3^ reported angina after transcatheter shunt closure due to extensive mural thrombosis in a residual 12 mm dilated coronary artery. Long-term anticoagulation therapy can be necessary even after surgery or intervention.^3^ Davis *et al*.^12^ reported the risk of thrombus formation in a blind pouch and distal embolism, recommending antiplatelet therapy. Therefore, Hijji *et al*.^13^ suggested that when the proximal coronary artery is enlarged to > 10 mm, fistula closure with concomitant bypass grafting to the distal artery is recommended to prevent intraluminal thrombosis.

Our patient had long, diffuse aneurysmal dilatation of the RCA. The surgical approach was planned with three objectives: Avoiding early and late complications. First, the shunt flow should be eliminated. The RCA was divided and closed at the proximal portion, distal to the conus branch originating from the RCA origin. Care was taken to avoid creating a blind pouch on the stump. The fistula was ligated distally to the posterior descending branch. The left circumflex arterial fistula was ligated at its origin. Second, the risk of late complications related to a dilated RCA, such as rupture, intracoronary thrombosis, and distal embolism, should be minimized. It was necessary to resect, depressurize, and isolate the dilated RCA from the distal coronary perfusion. Third, myocardial perfusion to the right ventricle was warranted, because Zhu *et al*.^14^ have demonstrated the clinical importance of postoperative right ventricular function and ventricular arrhythmia. For these reasons, the dilated RCA was replaced by a saphenous vein graft with anastomoses to two major right ventricular branches and a posterior descending artery. The orifices of these branches were trimmed into buttons that were anastomosed with holes created on the side and end of the saphenous vein. This button technique may be advantageous for adjusting the angle and length of small nonatherosclerotic branches, making it suitable even for young patients. Finally, coronary flow into the conus branch, two major right ventricular branches, and the posterior descending artery was normalized. Unfortunately, one small fistula from the circumflex artery remained but was clinically negligible.

In conclusion, surgical intervention, including fistula closure, critical coronary branch reconstruction, and valvular defect correction effectively improved abnormal coronary and systemic flow dynamics and reduced the risk of rupture, embolism, heart failure, and other complications. Given the rarity and complexity of such cases, further studies are required to explore the haemodynamic influences and long-term outcomes of various surgical approaches in order to optimize treatment strategies and enhance patient outcomes.

## Supplementary Material

ytaf269_Supplementary_Data

## Data Availability

The data relating to this publication are available in the article and online Supplementary material.

## Lead author biography

Yuya Yamazaki is a cardiovascular surgeon at Akita University Hospital, Akita, Japan. He completed his residency training in Akita University and Iwate Medical University Hospital, Iwate, Japan. He has a particular interest in coronary surgery, minimally invasive cardiac surgery, and congenital surgery.

## References

[1] Said SA, Lam J, van der Werf T. Solitary coronary artery fistulas: a congenital anomaly in children and adults. A contemporary review. Congenit Heart Dis 2006;1:63–76. 18377549 10.1111/j.1747-0803.2006.00012.x

[2] Li X, Song L, Guo H, Jin J, Xu M. Surgical repair of congenital coronary artery fistula with giant aneurysm. Heart Surg Forum 2020;23:E151–E153. 32364904 10.1532/hsf.2813

[3] Valente AM, Lock JE, Gauvreau K, Rodriguez-Huertas E, Joyce C, Armsby L, et al Predictors of long-term adverse outcomes in patients with congenital coronary artery fistulae. Circ Cardiovasc Interv 2010;3:134–139. 20332380 10.1161/CIRCINTERVENTIONS.109.883884

[4] Yun G, Nam TH, Chun EJ. Coronary artery fistulas: pathophysiology, imaging findings, and management. Radiographics 2018;38:688–703. 29601265 10.1148/rg.2018170158

[5] Mangukia CV . Coronary artery fistula. Ann Thorac Surg 2012;93:2084–2092. 22560322 10.1016/j.athoracsur.2012.01.114

[6] Firstenberg MS, Azoury F, Lytle BW, Thomas JD. Interposition vein graft for giant coronary aneurysm repair. Ann Thorac Surg 2000;70:1397–1398. 11081908 10.1016/s0003-4975(00)01579-4

[7] Ghaffari S, Akbarzadeh F, Pourafkari L. Aneurysmal coronary arteriovenous fistula closure with covered stent deployment: a case report and literature review. Cardiol J 2011;18:556–559. 21947993 10.5603/cj.2011.0013

[8] Wauthy P, Demanet H, Deuvaert FE. Surgical treatment of coronary artery fistula with aneurysm. Acta Chir Belg 2003;103:532–533. 14653046 10.1080/00015458.2003.11679485

[9] Thakkar B, Patel N, Poptani V, Madan T, Saluja T, Shukla A, et al Clinical and angiographic follow-up of coronary artery fistula interventions in children: techniques and classifications revisited. Cardiol Young 2015;25:670–680. 24775405 10.1017/S1047951114000614

[10] Bauer HH, Allmendinger PD, Flaherty J, Owlia D, Rossi MA, Chen C. Congenital coronary arteriovenous fistula: spontaneous rupture and cardiac tamponade. Ann Thorac Surg 1996;62:1521–1523. 8893601 10.1016/0003-4975(96)00757-6

[11] Nakahira A, Sasaki Y, Hirai H, Fukui T, Motoki M, Takahashi Y, et al Rupture of the aneurysmal circumflex coronary artery into the left atrium after ligation of the arteriovenous fistula. Circ J 2007;71:1996–1998. 18037761 10.1253/circj.71.1996

[12] Davis JT, Allen HD, Wheller JJ, Chan DP, Cohen DM, Teske DW, et al Coronary artery fistula in the pediatric age group: a 19-year institutional experience. Ann Thorac Surg 1994;58:760–763. 7944700 10.1016/0003-4975(94)90743-9

[13] Al-Hijji M, El Sabbagh A, El Hajj S, AlKhouli M, El Sabawi B, Cabalka A, et al Coronary artery fistulas indications, techniques, outcomes, and complications of transcatheter fistula closure. JACC Cardiovasc Interv 2021;14:1393–1406. 34238550 10.1016/j.jcin.2021.02.044

[14] Zhu F, Zheng Z, Yao L, Mou Y, Cheng Y, Gao H. Isolated right ventricular hypoplasia caused by a giant aneurysm of the right coronary artery to the left ventricular fistula in an adult: a case report. J Cardiothorac Surg 2016;11:93. 27377631 10.1186/s13019-016-0494-z PMC4932758

